# Application of Adaptive MOMEDA with Iterative Autocorrelation to Enhance Weak Features of Hoist Bearings

**DOI:** 10.3390/e23070789

**Published:** 2021-06-22

**Authors:** Tengyu Li, Ziming Kou, Juan Wu, Fen Yang

**Affiliations:** 1 College of Mechanical and Vehicle Engineering, Taiyuan University of Technology, Taiyuan 030024, China; litengyu0001@link.tyut.edu.cn (T.L.); wujuan@tyut.edu.cn (J.W.); 2 Shanxi Province Engineering Technology Research Center for Mine Fluid Control, Taiyuan 030024, China; 3 National-Local Joint Engineering Laboratory of Mining Fluid Control, Taiyuan 030024, China; 4 School of Mechanical Engineering, North University of China, Taiyuan 030051, China; nucmyf125@nuc.edu.cn

**Keywords:** low-speed hoist bearing, weak feature extraction, iterative autocorrelation, autocorrelation kurtosis entropy, multipoint optimal minimum entropy deconvolution adjusted

## Abstract

Low-speed hoist bearings are characterized by fault features that are weak and difficult to extract. Multipoint optimal minimum entropy deconvolution adjusted (MOMEDA) is an effective method for extracting periodic pulses in a signal. However, the decomposition effect of MOMEDA largely depends on the selected pulse period and filter length. To address these drawbacks of MOMEDA and accurately extract features from the vibration signal of a hoist bearing, an adaptive feature extraction method is proposed based on iterative autocorrelation (IAC) and MOMEDA. To automatically identify the pulse period, a new evaluation index named autocorrelation kurtosis entropy (AKE) was constructed to select the optimal IAC. To eliminate the influence of the filter length on the decomposition effect, an iterative MOMEDA strategy was designed to gradually enhance signal impulse features. The Case Western Reserve University bearing dataset and bearing data from a self-made hoisting test setup were used to verify the effectiveness of IAC-MOMEDA in extracting weak features. Moreover, the capability of IAC-MOMEDA for features extraction of normal bearing vibration signal was further confirmed by field test data.

## 1. Introduction

The multi-rope friction hoist is a key piece of equipment that connects surface and underground mining operations. The maximum hoisting height can exceed one kilometer, and the load acting on the head sheave can exceed one hundred tons. Once the bearing sustains initial damage, these heavy loads will cause the rapid development of further damage, which can quickly lead to serious economic losses and pose safety hazards. Therefore, studying the state detection of hoisting bearings is of great significance. However, at present, the on-site diagnosis of hoist bearings is still based on personal experience, and each inspection requires workers to climb a platform that is tens of meters in height, which makes it difficult to identify bearing faults before they cause damage [1].

Compared with ordinary bearings, hoist bearings are characterized by heavier loads and lower speeds [2]. A low rotation speed leads to weak fault vibration signals, and the vibration energy is easily attenuated in the transmission process. Moreover, the measured signal includes the vibrations of other mechanical components and the noise generated by the harsh working environment, which can mask a weak fault signal [3]. For the diagnosis of weak faults in bearings, intelligent classification methods based on machine learning and feature frequency extraction methods based on signal processing are both effective approaches.

Intelligent classification methods do not require the user to have extensive background knowledge or experience related to signals. These approaches enable automatic diagnosis through complex algorithms, such as boosted tree [4], support vector machine [5], and sparse autoencoder [6], and they perform well in a laboratory environment. Intelligent classification methods are currently a research hotspot, but acquiring a large amount of field data on hoist bearing faults is difficult for safety reasons, so further research is necessary for their practical application [7].

Bearing feature extraction has been the focus of many studies and has a variety of applications, but determining how to extract effective features from weak fault signals is still challenging. Because of the nonlinear and nonstationary characteristics of vibration signals, signal decomposition techniques [8,9,10,11,12] can effectively eliminate noise and extract fault features. However, these methods also have shortcomings, such as mode aliasing and weakening useful signal features [13]. The main basis of fault detection is the generation of periodic impact components when a bearing fails. Blind deconvolution (BD) techniques extract periodic pulse components from the measured signal by designing filters for fault diagnosis. Minimum entropy deconvolution (MED) is a typical BD method. Yang et al. combined MED and a signal decomposition method to extract features from the vibration signal of a hoist bearing [14]. In MED, kurtosis is used as the objective function to iteratively identify the optimal inverse filter. Kurtosis is sensitive to random pulses, which can easily cause errors. As an improved method of MED, maximum correlated kurtosis deconvolution (MCKD) was proposed. Although MCKD can effectively enhance periodic pulse signals, it requires the a priori impact period and resampling.

Motivated by the above research, multipoint optimal minimum entropy deconvolution adjusted (MOMEDA) was proposed [15]. This method uses a time target vector to determine the position and weight of the impulse and obtains the optimal filter without iteration by maximizing the multi-D-norm (MDN). Because it is effective in extracting periodic shocks, the MOMEDA method has been used for weak feature extraction [16,17]. However, determining the impulse repetition period a priori has always been a difficult problem in MOMEDA. Currently, there are two common methods for pre-selecting the signal period. The first is to construct a suitable multi-objective optimization function and then use optimization algorithms such as grid search [18], grasshopper optimization algorithm [19], or particle swarm optimization [20] to identify the optimal period and filter length. However, these methods often require dozens of iterations, which is computationally complex and time-consuming. Another method is period detection technology (PDT). The autocorrelation function (ACF) is a common PDT [21]. Compared with multipoint kurtosis (Mkurt), indicator of cyclostationarity (ICS), and MDN, the ACF has the advantages of simple calculation and easy automation [22]. Periodic modulation intensity (PMI) is an improved method based on the ACF, as it can further eliminate the noise that remains with the ACF [23]. However, in the presence of complex background noise, it is difficult to completely eliminate noise by a single application of the ACF or PMI. Cheng et al. performed iteration PMI-MOMEDA to update the signal period and enhance the effect, but this method still heavily relies on the signal period extracted from the first PMI [24]. Zhang et al. proposed an iterative autocorrelation (IAC) denoising method [25], and Pang et al. used IAC to process the envelope spectrum obtained by TEO to enhance the periodic features of the signal [26]. However, these studies did not present a method to automatically select the number of iterations for a signal with unknown states.

Based on the above analysis, this paper proposes an objective function to select the optimal ACF for identifying the signal period in the IAC process, and uses an iterative MOMEDA strategy to eliminate the influence of filter length on the deconvolution effect. Finally, the weak periodic pulse components of the signal are accurately extracted. Section 2 of this article introduces the basic theories underlying MOMEDA, IAC, and the constructed indicator. Section 3 shows the steps of the proposed method (IAC-MOMEDA). In Section 4, IAC-MOMEDA is explained and analyzed in detail, and its effectiveness is verified by experimental data. Section 5 presents the conclusions.

## 2. Basic Theory of the Proposed Technique

### 2.1. MOMEDA

The impact signal *y*(*t*) generated by the bearing fault is modulated by the transfer function *h*(*t*) as *s*(*t*), and, together with noise *n*(*t*), is collected by the sensor to form the measurement signal *x*(*t*). The process can be expressed as follows [27]:

(1) x(t)=s(t)+n(t)=h(t)∗y(t)+n(t)

The goal of MOMEDA is to design an inverse filter ***f*** to extract the impulse signal:

(2) y=f∗x

In order to achieve this goal, a target vector ***t*** composed of constants is constructed according to the signal period to describe the weight and position of the impact pulse [28]. Then, multipoint D-norm (MDN) is defined to reflect the impulse characteristics of the filtered signal:

(3) $$\mathrm{MDN}=\frac{1}{\left\|t\right\|}\frac{t^{T}y}{\left\|y\right\|}$$

The maximization problem of MDN is the solution to the optimal inverse filter:

(4) $$\max\;\mathrm{MDN}=\underset{f}{\max}\frac{t^{T}y}{\left\|y\right\|}$$

Then, the filter and output solution of MOMEDA can be generalized as follows:

(5) $$f={\left(X_{0}X_{0}{}^{T}\right)}^{-1}X_{0}t$$

(6) $$X_{0}=\left[\begin{array}{ccccc} x_{L} & x_{L+1} & x_{L+2} & \cdots & x_{N}\\ x_{L-1} & x_{L} & x_{L+1} & \cdots & x_{N-1}\\ x_{L-2} & x_{L-1} & x_{L} & \cdots & x_{N-2}\\ \vdots & \vdots & \vdots & & \vdots \\ x_{1} & x_{2} & x_{3} & \cdots & x_{N-L+1} \end{array}\right]$$

(7) $$y=X_{0}{}^{T}f$$

where ***X***_0_ is the sampling matrix determined by the input signal ***x***.

MOMEDA can effectively extract the periodic signal shock in the signal, and reduce the impact of random shock. However, in practice, the signal period is usually unknown, which greatly limits the use of MOMEDA. Selecting the wrong parameters will enhance the false impact signal, leading to misdiagnosis. Therefore, the accurate selection of the signal period is a key issue in MOMEDA and can significantly improve its application value.

### 2.2. Iterative Autocorrelation

The autocorrelation function (ACF) is an effective signal period extraction method that can strengthen the signal period while retaining the original signal information. The ACF of the vibration signal *x*(*t*) can be expressed by Equation (8):

(8) $$R_{xx}\left(\tau_{1}\right)=\int_{-\infty }^{\infty }x\left(t+\tau_{1}\right)x\left(t\right)dt$$

where *τ*_1_ is the time delay. Substituting Equation (1) into the above equation, the result is as follows:

(9) $$\begin{array}{ll} R_{xx}\left(\tau_{1}\right) & =\int_{-\infty }^{\infty }s\left(t+\tau_{1}\right)s\left(t\right)dt+\int_{-\infty }^{\infty }s\left(t+\tau_{1}\right)n\left(t\right)dt+\int_{-\infty }^{\infty }n\left(t+\tau_{1}\right)s\left(t\right)dt+\int_{-\infty }^{\infty }n\left(t+\tau_{1}\right)n\left(t\right)dt\\ & =R_{ss}\left(\tau_{1}\right)+R_{sn}\left(\tau_{1}\right)+R_{ns}\left(\tau_{1}\right)+R_{nn}\left(\tau_{1}\right) \end{array}$$

where *R_ss_*(*τ*_1_) and *R_nn_*(*τ*_1_) are the ACF of the periodic pulse signal *s*(*t*) and noise *n*(*t*), respectively, and *R_sn_*(*τ*_1_) and *R_ns_*(*τ*_1_) denote the cross-correlation of *s*(*t*) and *n*(*t*). Since *n*(*t*) is white noise and is completely independent of *s*(*t*), *R_sn_*(*τ*_1_), *R_ns_*(*τ*_1_), and *R_nn_*(*τ*_1_) are all 0, theoretically. Then, *R_xx_*(*τ*_1_) *≈ R_ss_*(*τ*_1_), the noise is completely eliminated, and the periodic component of the vibration signal is enhanced. However, in a practical situation, the noise component is complex, and it is difficult to completely eliminate it using the ACF [26]. On this basis, iterative autocorrelation (IAC) is proposed and described as:

(10) $$R_{xx}{}^{k}\left(\tau_{1}\right)=\int_{-\infty }^{\infty }R_{xx}{}^{k-1}\left(t+\tau_{1}\right)R_{xx}{}^{k-1}\left(t\right)dt\;\;\left(k\geq 2\right)$$

where *k* is the number of iterations. Previous studies have shown that the appropriate number of iterations can make IAC reduce the signal noise better. The greater the number of iterations, the more significant the denoising effect, and the closer the signal is to the sine curve. However, more iterations expand the resonance active range and obscure the signal period characteristics [25]. Therefore, choosing the appropriate number of iterations is a key step in strengthening the signal period.

### 2.3. Construction of Autocorrelation Kurtosis Entropy Index

The regularity of a time series can be quantified by discrete entropy (DE), which is characterized by less calculation time, excellent anti-interference capability, and high sensitivity to changes in frequency, amplitude, and bandwidth [29]. Its mathematical expression is as follows:

(11) $$DE\left(c,m,\tau_{2}\right)=-\sum_{i=1}^{c^{m}}P\left(\pi_{v\left(i\right)}\right)\mathrm{In}\left(P\left(\pi_{v\left(i\right)}\right)\right)$$

where *π_v_*_(*i*)_ represents different dispersion patterns, *P*(*π_v_*_(*i*)_) is the relative frequency of each dispersion pattern, *c* is the number of classes, *m* is the embedding dimension and *τ*_2_ is the time delay.

Compared with the use of regular or entropy indicators alone, the combination of the two can more accurately reflect the operating status of low-speed bearings [30,31]. IAC can effectively eliminate noise and reduce the influence of random shocks on kurtosis. Therefore, the autocorrelation kurtosis entropy (AKE) composite index was constructed by combining the normalized exponential function of kurtosis and DE, that is:

(12) $$AKE\left(n\right)=\frac{\exp\left(P\left(kurt_{n}\right)\right)/\sum \exp\left(P\left(kurt\right)\right)}{\exp\left(P\left(DE_{n}\right)\right)/\sum \exp\left(P\left(DE\right)\right)}$$

where *P*(*kurt_n_*) and *P*(*DE_n_*) are the percentages of the *n*th kurtosis and DE values, respectively. When the periodic shock component can be clearly reflected by the optimal IAC, the kurtosis will increase and the DE will decrease. Therefore, the AKE index can effectively reflect the significance of the periodic shock component characteristics.

## 3. Algorithm Flow of Adaptive MOMEDA with IAC

**Figure 1:**
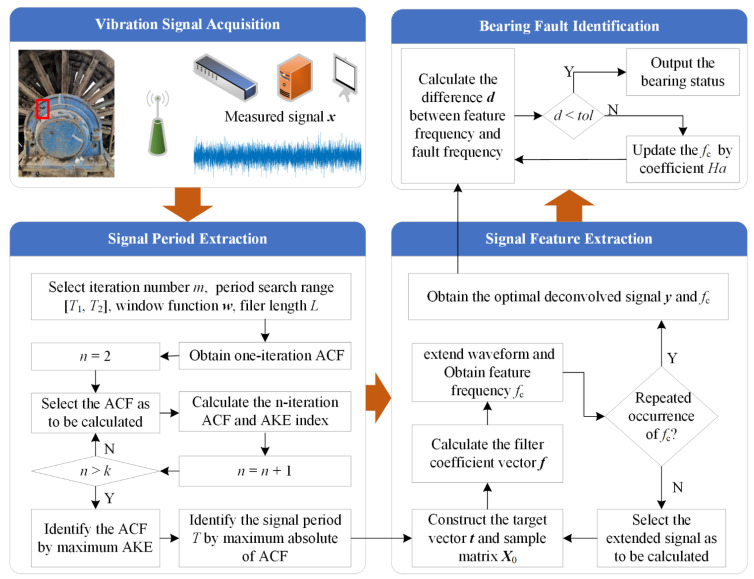
Algorithm flow of adaptive MOMEDA with IAC.

The flowchart of the proposed algorithm is shown in Figure 1. The step 1 is to extract the raw signal period based on IAC and AKE. The AKE indexes are used to choose optimal IAC, and the signal period is determined by maximum absolute amplitude in the optimal IAC. The specific process is shown in Algorithm 1:
**Algorithm 1** Signal Period Extraction.  **Input**: Measured signal ***x***     Period search range [*T*_1_, *T*_2_]     Maximum number of iterations *k*     Threshold valued *thre* = 0  **Output**: Signal period *T*  Initialize the input parameters;  **for**
*i* = 1 to *k*  ***xx**_i_* = ACF(***x***)         Compute the ACF of ***x***, Equation (8)  *ind_i_* = AKE(***xx**_i_*(*T*_1_:*T*_2_))    Compute the AKE of ACF, Equation (12)  ***x*** = ***xx**_i_*  **if**
*i* > 1   **if**
*ind_i_* > *thre*       *j* = *i*          Obtain the optimal number of iterations      *thre* = *ind_i_*   **end if**  **end if**  **end**  [~, *T*] = max(***xx**_j_*( *T*_1_:*T*_2_))       Identify the signal period *T*

Step 2 is the iterative MOMEDA processing based on signal period *T* to extract feature frequency. Signal waveform extension method [24] is applied to address the problem of the reduced length of the deconvolved signal obtained after MOMEDA. The extended signal is regarded as a new signal to be decomposed by MOMEDA until the same characteristic frequency reappears. The specific process is shown in Algorithm 2:
**Algorithm 2** Signal Feature Extraction.  **Input**: Measured signal ***x***     Signal period *T*     Feature frequency set ***fn*** = [0]     Cycle judgment index *m* = 1  **Output:** Feature frequency *f_c_*  Construct the target vector ***t*** through *T*;  **While**
*m* ≠ 0     ***y*** = MOMEDA(***x***)  Equation (3)–(7)     ***s*** = extension(***y***)    extend waveform [24]     ***Amp*** = FFT(***s***)     Obtain the envelope spectrum     *f_c_* = max(***Amp***)    Obtain the feature frequency *f_c_*     ***fd*** = ***fn*** − *f_c_*     *m* = min(abs(***fd***))  Feature frequency repetition recognition     ***fn*** = [***fn***
*f_c_*]     ***x*** = ***s***  **end**

Step 3 is to automatically identify the bearing state according to the difference between the extracted feature frequency and fault frequencies. By using the coefficient *Ha*, the problem that the characteristic frequency does not match the fault frequencies when the extracted signal period is a multiple of the theoretical fault period is solved. The specific process is shown in Algorithm 3:
**Algorithm 3** Bearing fault identification.  **Input:** Feature frequency *f_c_*     Fault frequency set ***f**_d_* = [*f_i_ f_o_ f_b_ f_r_*]     Frequency tolerance *tol*  **Output**: Bearing status ***d***  **for**
*Ha* = 1 *to* 5  ***d*** = abs(***f**_d_* − *f_c_* × *Ha*)  **if** min(***d***) < *tol*
   break  **end if**  **end**

**Figure 2:**
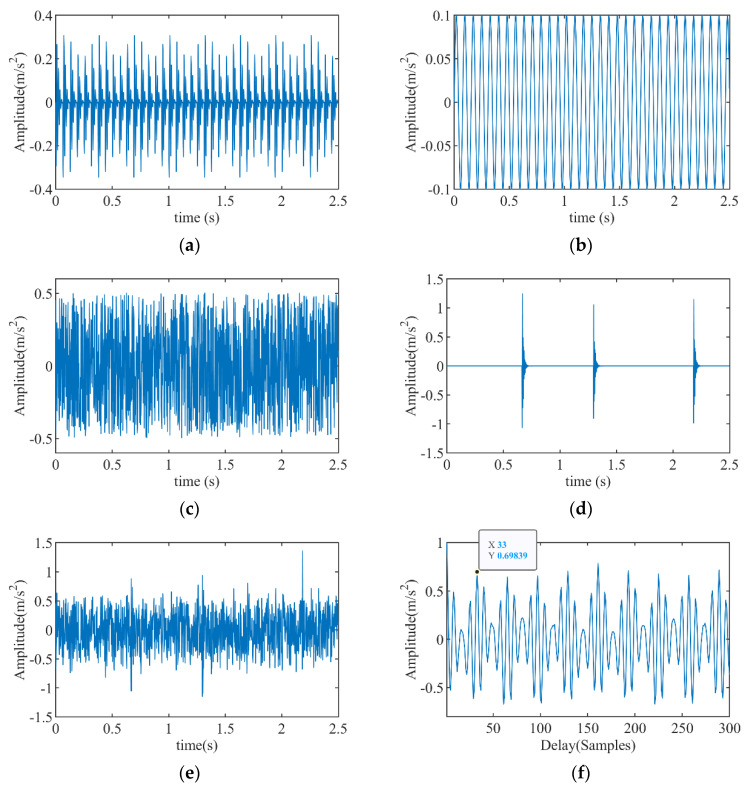
Simulation signals and result of period extraction: (**a**) bearing fault signal, (**b**) sine signal, (**c**) random signal, (**d**) non-periodic impulses, (**e**) synthetic signal, (**f**) IAC spectrum of synthetic signal.

In order to select the signal period accurately, it is necessary to analyze the anti-interference ability of the propose IAC method. The simulation signals and processing result are shown in Figure 2. Figure 2a shows the bearing fault signal, the fault period is *T* = 32. Figure 2b is cyclostationary signal indicating another fault or interference, and its period is *T* = 40. Figure 2c displays a random signal and Figure 2d gives the non-periodic impulses, both of them have larger amplitudes than fault signal. Figure 2e displays the synthetic signal formed by all interferences. Figure 2f shows the IAC spectrum of synthetic signal obtained by the proposed IAC method, it can be seen that the peak value at *T* = 33 is clearly prominent and close to the fault period of 32. This indicates that the proposed IAC method can effectively eliminate the influence of random and aperiodic shock signals, but it can’t extract all fault periods from a composite fault signal.

## 4. Experimental and Comparative Analysis

In this study, the effectiveness of the proposed algorithm was verified by using the Case Western Reserve University (CWRU) bearing dataset [32], bearing data from a self-made hoisting test setup, and field data from a head sheave.

### 4.1. Case 1: CWRU Data Analysis

**Figure 3:**
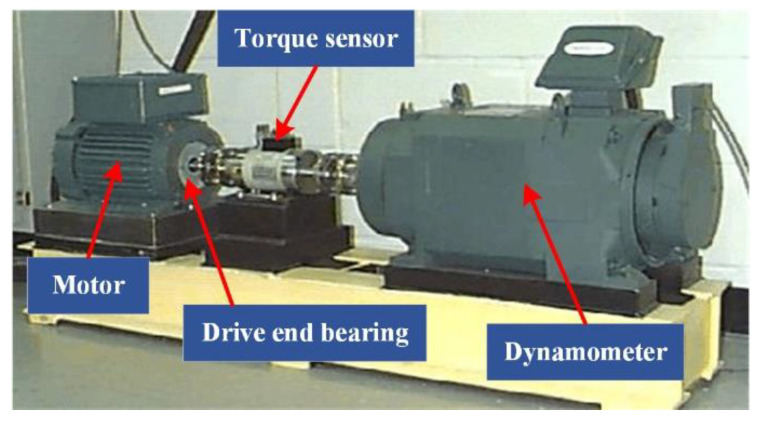
Bearing test bench in the CWRU.

The test bench is shown in Figure 3, which is mainly composed of a motor, torque sensor, dynamometer, and control electronics. Single point faults with a diameter of 7 mils were machined on the bearing to simulate different types of faults. The sensors are installed on the motor housing. The rotational frequency of the motor is 29.95 Hz, and the sampling frequency is 12,000 Hz. According to the bearing parameters, the theoretical fault frequencies of the inner ring, outer ring, and rolling element are 162.18, 107.36, and 141.17 Hz, respectively [19].

#### 4.1.1. Feature Extraction and Comparative Analysis of the Inner Ring Fault Signal

The proposed algorithm parameters were initialized according to the characteristics of the vibration signal. The theoretical periods corresponding to the rotational frequency and the inner fault frequency are 400.66 and 73.99, respectively, so the signal period search range is set to [70, 420]. The number of iterations is *k* = 9, the filter length is *L* = 1000, and the window function is ***w*** = [1 1 1 1 1], and the frequency tolerance *tol* = 3.

**Figure 4:**
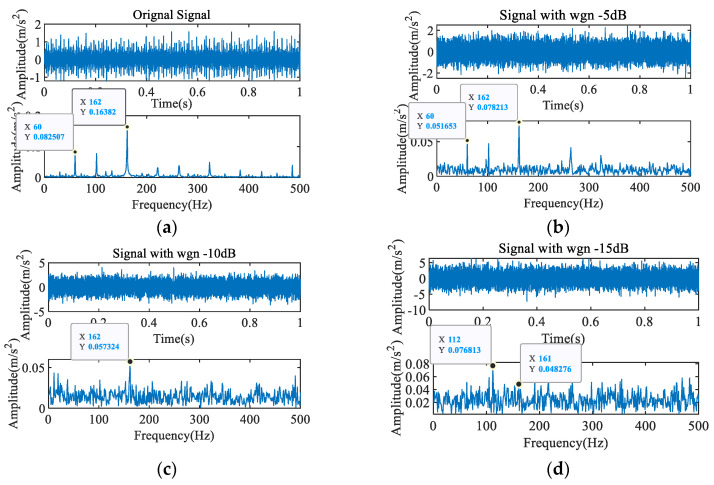
Inner ring fault signal with different intensities of noise.

**Figure 5:**
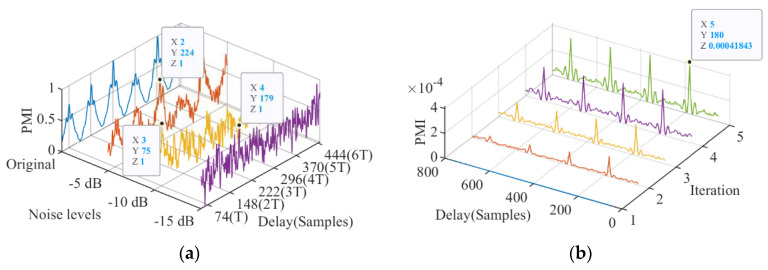
Identification of periodic shocks based on PMI: (**a**) PMI spectra with different noise levels, (**b**) iterative PMI-MOMEDA spectra with SNR = −15 dB.

In order to accurately select the signal impulse period, the noise suppression performance of the proposed method was analyzed and compared with PMI. Figure 4 shows the mixed signals obtained by adding noise with different intensities to the original signal. The results show that as the noise intensity increases, the interference component in the envelope spectrum gradually strengthens. When the signal-to-noise ratio (SNR) is −15 dB, the periodic impact caused by the fault in the time domain and the fault frequency in the envelope spectrum are completely submerged. Figure 5 shows the results of using PMI to identify the impact period of the mixed signal. Figure 5a reveals that the greater the intensity of the added noise, the greater the effect on PMI, and when SNR = −15 dB, the period cannot be identified. The iterative PMI-MOMEDA method [24] was used to process the mixed signal with SNR = −15 dB, and the result is shown in Figure 5b. Because the wrong period is extracted first, the false impact component is strengthened in each iteration, and it is difficult to correct the period.

**Figure 6:**
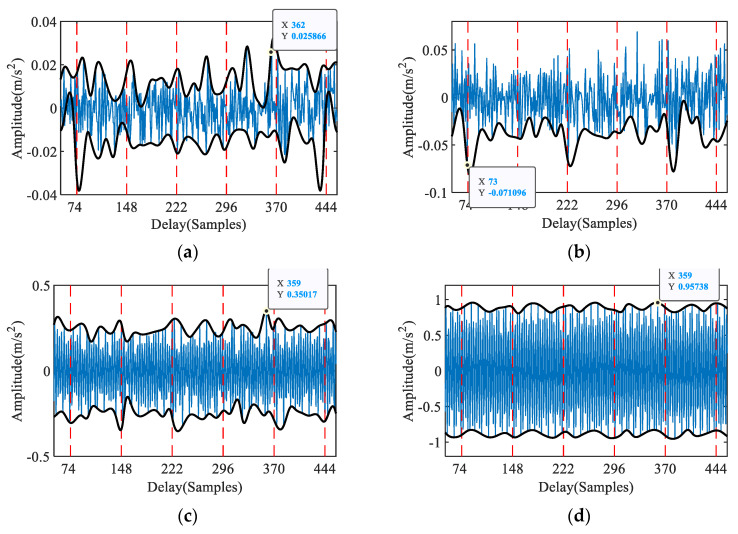

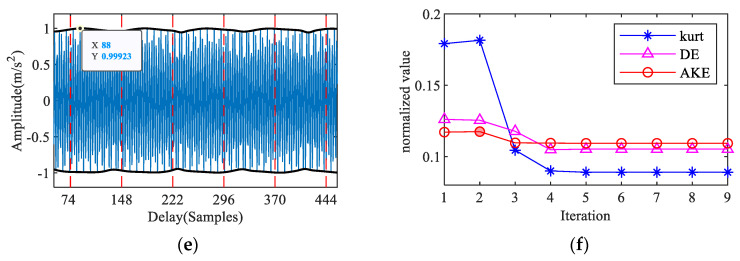
Period identification based on IAC: (**a**) 1st iteration (ACF), (**b**) 2nd iteration, (**c**) 3rd iteration, (**d**) 4th iteration, (**e**) 5th iteration, (**f**) AKE index.

As a comparison, Figure 6 shows the periodic identification results of the proposed method. Figure 6a–f display the 1st–5th IAC, respectively. The red dashed line represents the theoretical fault periods, and the black solid line represents the period of the IAC. As the number of iterations increases, a period becomes evident in the second IAC, and the cyclic characteristics of the subsequent iterative curves gradually disappear. The AKE index generally reflects these changes, reaching the maximum in the second iteration and then decreasing, as shown in Figure 6f. In the second IAC, the maximum absolute amplitude is at *T* = 73, which is close to the theoretical inner ring fault period of 73.99. These results show that the proposed IAC can accurately extract the fault period from complex noise.

**Figure 7:**
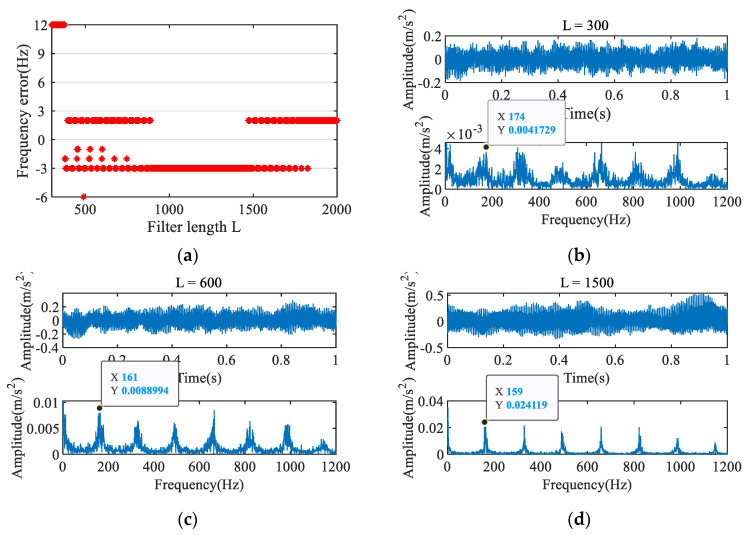
Influence of different filter lengths on feature frequency extraction.

For MOMEDA, filter length is also a very critical parameter. To illustrate the influence of filter length, MOMEDA was used to process the signal with filters of different lengths. The filter length range is set to [300, 2000], and the step size is 1. The results are shown in Figure 7. Figure 7a shows the difference between the extracted characteristic frequency and the theoretical fault frequency for each filter length. Most of the extracted characteristic frequencies are concentrated near the fault frequency, indicating that the extracted characteristic frequency is mainly affected by the fault period, but an inappropriate filter length will still lead to the extraction of the wrong characteristic frequency. In Figure 7b–d, as the filter length increases, the impact components become more visible, the interference frequency in the envelope spectrum is greatly reduced, and the characteristic frequency becomes more prominent. However, the filter length does not have a direct relationship with the accuracy of the characteristic frequency relative to the actual fault frequency.

**Figure 8:**
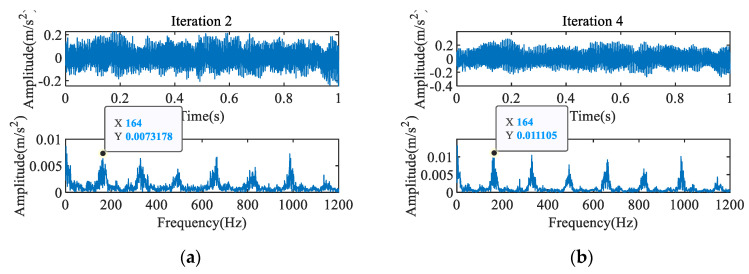
Results of iterative MOMEDA.

To avoid diagnostic errors caused by unsuitable filter lengths, iterative MOMEDA is proposed. The filter length is set to L = 300, and the characteristic frequency extracted by MOMEDA is 174 Hz, as shown in Figure 7b. The impact component of the signal obtained in the second iteration is enhanced, and the corresponding characteristic frequency is 164 Hz, the noise is significantly reduced, as shown in Figure 8a. After the fourth iteration, the characteristic frequency of 164 Hz recurs, and the impulse component and noise reduction are further enhanced, as shown in Figure 8b. According to algorithm 3, it can be determined that the characteristic frequency of 164 Hz represents the inner ring fault. These results show that the iterative MOMEDA method can effectively eliminate the influence of the filter length on the final result to obtain an accurate fault frequency.

#### 4.1.2. Feature Extraction of Other Bearing States

**Figure 9:**
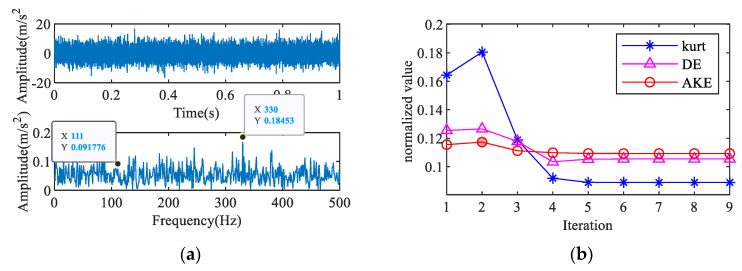

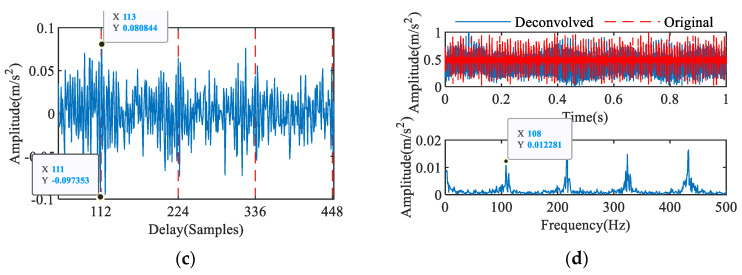
Feature extraction of outer ring fault signal: (**a**) signal with noise, (**b**) AKE index, (**c**) 2nd IAC, (**d**) 3rd iterative MOMEDA.

**Figure 10:**
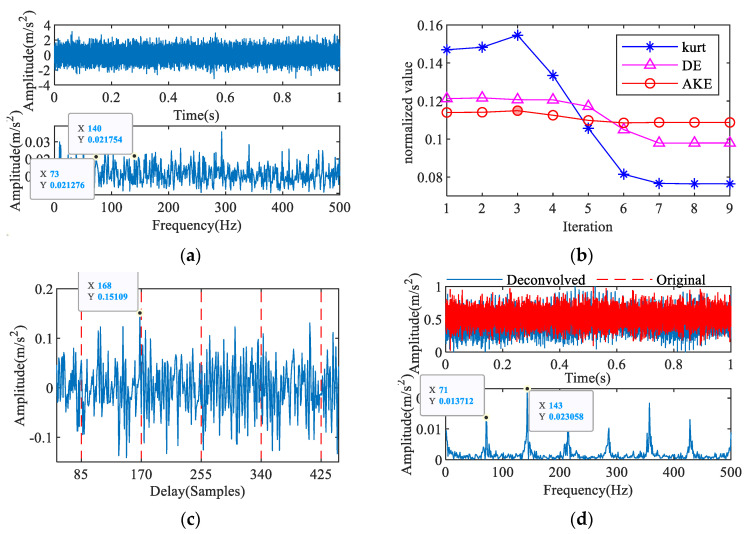
Feature extraction of rolling element fault signal: (**a**) signal with noise, (**b**) AKE index, (**c**) 3rd IAC, (**d**) 2nd iterative MOMEDA.

The feature extraction result of the outer ring fault signal with a noise intensity of -15 dB is shown in Figure 9. Figure 9a shows the time-domain waveform and envelope spectrum of the mixed signal. The fault frequency is masked by the noise and is difficult to identify. Figure 9b–c show the results of fault period extraction based on IAC. The results show that the second IAC has a certain periodicity, and the maximum absolute amplitude appears at *T* = 111, which is close to the theoretical fault period. Figure 9d shows the comparison between the deconvolved signal and the original signal. The impact components in the deconvolved signal are very close to the raw signal, and a feature frequency of 108 Hz that corresponds to the outer ring fault is clearly reflected in the envelope spectrum. Figure 10 is the result of the fault diagnosis of the rolling element; the identified fault period is twice the ideal period, and characteristic frequencies of 71 and 143 Hz are extracted. According to algorithm 3, the fault of bearing rolling element can be diagnosed automatically.

**Figure 11:**
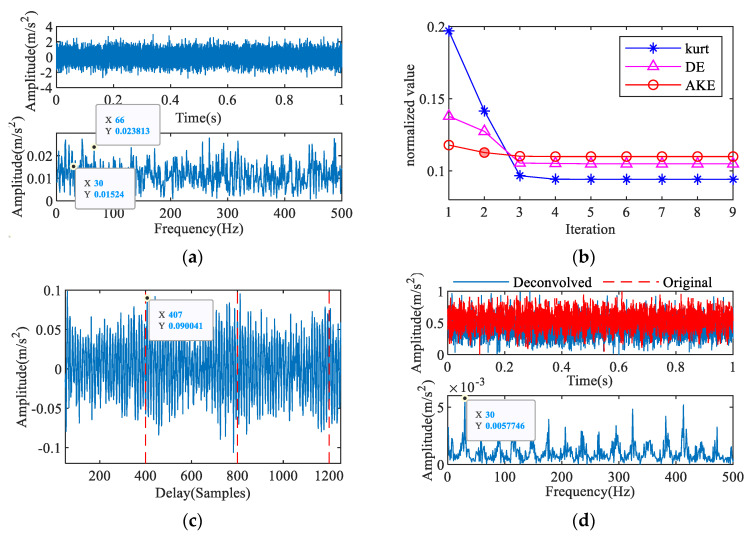
Feature extraction of normal signal: (**a**) signal with noise, (**b**) AKE index, (**c**) 2nd IAC, (**d**) 2nd iterative MOMEDA.

Bearings typically remain in a normal state for a long time in practical applications, so effectively identifying the normal state of the bearing can prevent a large number of misdiagnoses. The proposed IAC can effectively identify the period of the normal signal to extract the characteristic frequency, as shown in Figure 11. The AKE indices are shown in Figure 11b. The largest AKE appears in the second iteration of iterations 2–9, and the corresponding IAC is shown in Figure 11c. There is clear periodicity in the curve, and the maximum absolute value of the amplitude is at *T* = 407. In Figure 11d, the characteristic frequency obtained by iterative MOMEDA is 30 Hz, which is close to the rotation frequency.

### 4.2. Case 2: Bearing Data Analysis of Self-Made Hoisting Testing Setup

**Figure 12:**
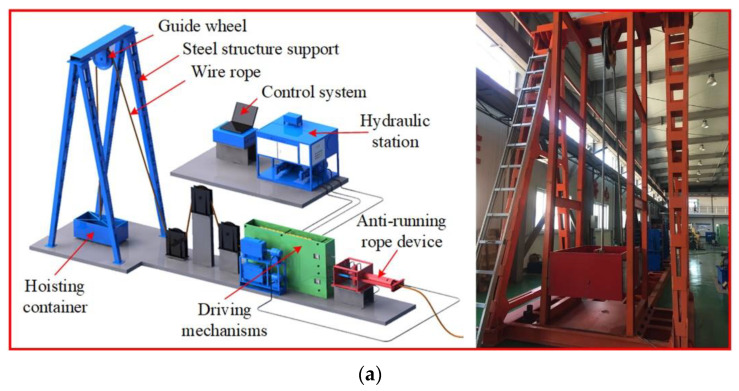

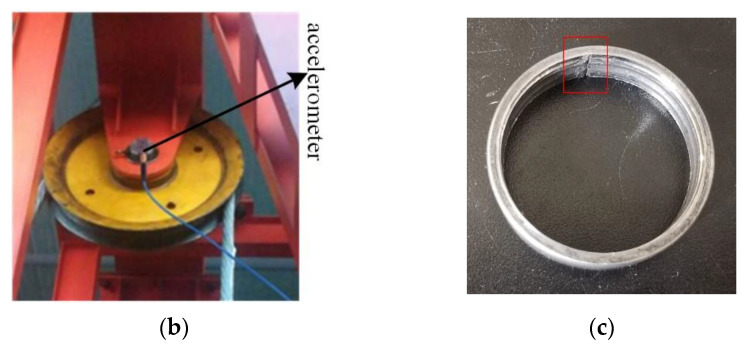
Simulation test of bearing fault diagnosis: (**a**) self-made hoisting test setup, (**b**) fixed sensor position, (**c**) bearing outer ring fault.

The self-made hoisting test setup is shown in Figure 12a, which is mainly composed of driving mechanisms, steel wire rope, guide wheel, container, and steel structure support. A deep groove ball bearing (SKF6203) was selected as the research object, and the sensor was fixed on the bearing seat of the guide wheel to collect the vibration signals of the bearing outer ring fault and the normal state, as shown in Figure 12b. The fault of the bearing outer ring was simulated by manually adding a scratch with a width of 1 mm and a depth of 0.5 mm, as shown in Figure 12c. The rotation frequency is about 3.2 Hz, the sampling frequency is 1280 Hz, and the theoretical outer ring fault frequency is 9.79 Hz. The filter length is set to 500, and the other parameters are the same as in Case 1.

**Figure 13:**
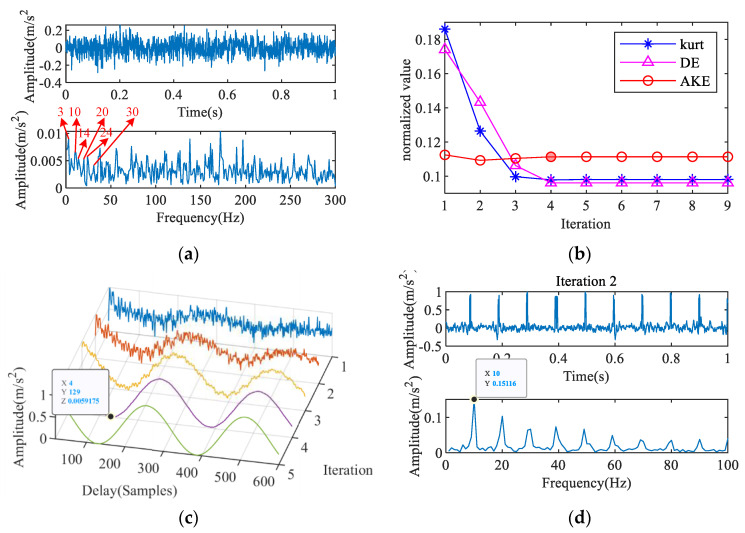
Feature extraction of outer ring fault signal: (**a**) measured signal, (**b**) AKE index, (**c**) 4th IAC, (**d**) 2nd iterative MOMEDA.

**Figure 14:**
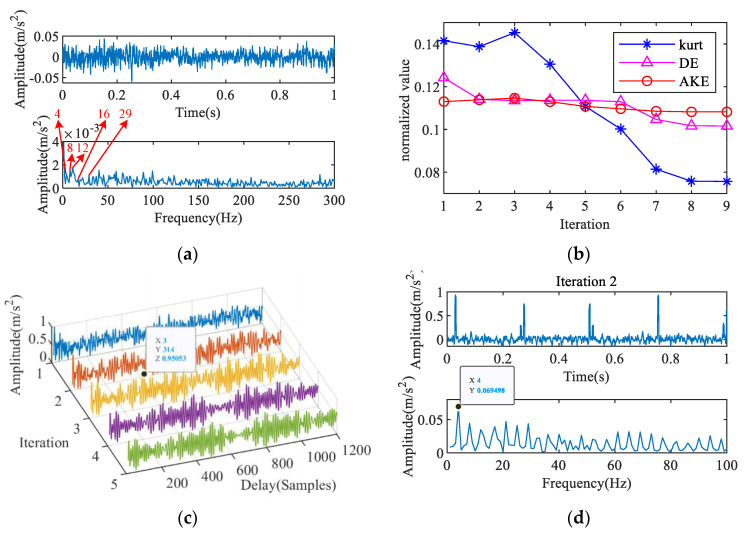
Feature extraction of normal signal: (**a**) measured signal, (**b**) AKE index, (**c**) 3rd IAC, (**d**) 2nd iterative MOMEDA.

The collected signal of the outer ring fault is shown in Figure 13a, and a rotational frequency of 3 Hz, a feature frequency of 10 Hz close to outer ring fault frequency of 9.79 Hz, and its associated harmonics, and side frequencies are visible in the envelope spectrum. According to Figure 13b,c, the fourth IAC is the optimal iteration result, and the fault period is *T* = 129. The extracted features are shown in Figure 13d. The impact components in the time domain are enhanced, and the maximum amplitude in the envelope spectrum appears at a feature frequency of 10 Hz. The diagnosis result of the normal signal is shown in Figure 14; the extracted characteristic frequency is 4 Hz, which is close to the rotational frequency.

### 4.3. Case 3: On-Site Hoisting Bearing State Detection

**Figure 15:**
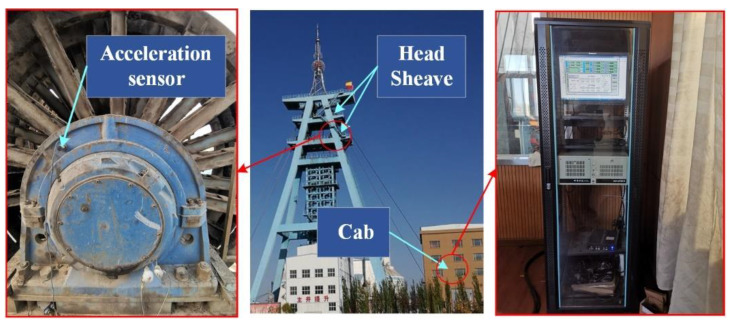
State detection of head sheave bearing.

The maximum speed of the hoist is 10.89 m/s, the diameter of the head sheave is 4 m, and the rotation frequency is 0.87 Hz. The status of bearing SKF241/630 CAK/W33 is normal, and there are no other faults. The sampling frequency is 256 Hz, and the theoretical period corresponding to the normal state is 294.25. The period search range is set to [20 350], and the other parameters remain unchanged. The installation position of the three-way accelerometer is shown in Figure 15, and the data is transmitted wirelessly to the computer in the driver’s cab. In engineering practice, considering the installation and maintenance of equipment, real-time signal and other factors, the field hopes to obtain more accurate results through fewer sensors, so one sensor is used to collect the data of a single bearing in the experiment.

**Figure 16:**
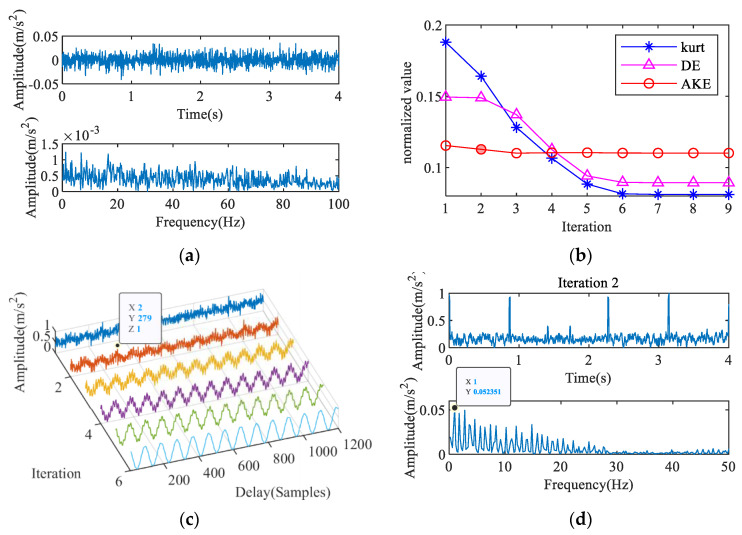
Feature extraction of field signal: (**a**) measured signal, (**b**) AKE index, (**c**) 2nd IAC, (**d**) 2nd iterative MOMEDA.

The collected vibration signal is shown in Figure 16a. Some shock components can be seen in the time-domain waveform, and there is no clear characteristic frequency in the envelope spectrum. Figure 16b,c show the IAC and its corresponding AKE index. The figure reveals that the maximum absolute value of the amplitude is at *T* = 279. Figure 16d shows the deconvolved signal waveform and its envelope spectrum. According to these results, the characteristic frequency extracted is 1 Hz, which is close to the rotation frequency. This indicates that the bearing is free from failure and is consistent with the preliminary results.

## 5. Conclusions

In this work, an IAC-MOMEDA method is proposed for extracting the weak features of bearing vibration signals. The following main conclusions are obtained:

(1) The decomposition accuracy of MOMEDA is affected by the signal period and the filter length. The characteristic frequency is mainly affected by the period, and the wrong signal period can lead to the enhancement of false pulse components. However, even if the fault period is accurate, an inappropriate filter length may still cause the extraction of the wrong characteristic frequency.
(2) The AKE index is introduced to IAC for the automatic identification of the signal period. The proposed method is robust to a complex noise background. The proposed iterative MOMEDA method can effectively eliminate the influence of filter length on the final effect.
(3) The proposed method is verified by multiple sets of test data, and the results show that the proposed method can accurately identify faults and the normal state. Analysis using field data shows that the proposed method can effectively diagnose the working state of a hoisting bearing.

In further work, the influence of other parameters will be researched to improve the robustness of the proposed method, and IAC-MOMEDA and signal decomposition technology will be combined to accurately diagnose the multiple defects bearing.

## Data Availability

The data used to support the findings of this study are available from the corresponding author upon request.
